# Evaluation of Engineering Properties of Calcium Sulfoaluminate Cement-based Concretes Reinforced with Different Types of Fibers

**DOI:** 10.3390/ma12132151

**Published:** 2019-07-04

**Authors:** Vahid Afroughsabet, Luigi Biolzi, Sara Cattaneo

**Affiliations:** 1 Department of Architecture, Built Environment and Construction Engineering, Politecnico di Milano, 20133 Milan, Italy; 2 Department of Civil and Mineral Engineering, University of Toronto, Toronto, ON M5S 1A4, Canada; 3 Construction Technologies Institute, Italian National Research Council (ITC-CNR), 20098 San Giuliano Milanese (MI), Italy

**Keywords:** calcium sulfoaluminate cement, fiber-reinforced concrete, double hooked-end steel fibers, PVA fibers, mechanical properties, dimensional stability

## Abstract

Calcium sulfoaluminate (CSA) cement has recently gained increased attention due to its lower amount of CO_2_ emissions, as compared to that of the ordinary Portland cement (OPC). This paper evaluates the impact of different types of fibers on the engineering features of CSA-based concretes at different water-cement ratios of 0.35 and 0.28. In this study, metallic fibers including double hooked-end steel fibers and hooked-end steel fibers, and non-metallic fibers (i.e., polyvinyl alcohol (PVA) fibers) were utilized at fiber content of 1%. The mechanical properties of concretes were assessed at different curing ages. Dimensional stability of the concrete mixes was also examined. The morphology of the fractured specimens was studied by using the SEM method. The results indicate that the engineering properties of concrete were improved by introducing fibers to the concrete, irrespective of fiber type. The results show that DHE steel fiber has an important effect on the flexural performance of CSA cement-based concretes and results in deflection-hardening behavior. It was observed that fibers and particularly PVA fibers cause a decrease in shrinkage deformation. Microstructure tests demonstrate that prismatic ettringite is the main hydration product of CSA cement-based concrete. The SEM observation also confirms that the inclusion of CSA cement in concrete improves the cohesiveness between the fibers and cement matrix.

## 1. Introduction

Portland cement concrete is utilized for different kinds of infrastructures and its huge amount of consumption over the world make it the most used material on the planet [1,2]. Conventional concrete is a composite that containing ordinary Portland cement (OPC), aggregate, and water at proportions of approximately 12%, 80%, and 8%, respectively [3]. OPC is not considered an eco-friendly material because its production results in a significant fraction of the overall CO_2_ emissions [4]. Using alternative binders such as calcium sulfoaluminate (CSA), cement can be a promising solution to reducing the amount of CO_2_ emissions [5]. CSA cements were first developed by Alexander Klein at the University of California at Berkeley in the 1960s [6]. Thereafter, various kind of CSA cements were successfully produced in China in the 1970s and categorized as the “third cement series” [7,8]. CSA cements have several applications based on their performance, and this binder has been successfully used in China for the production of concrete in concrete pipes, bridges, precast concrete, waterproof layers, pre-stressed concrete, shotcrete, and low temperature constructions [9,10,11].

The main constituent of CSA clinker is ye’elimite (C_4_A_3_$), and depending on the raw material composition, it may contain minor phases like belite (C_2_S), calcium sulfate (C$), and aluminoferrite (C_4_AF) [12,13,14]. The increase in the content of ye’elimite in the CSA clinker leads to a higher strength in the early ages, while a raise in the belite content results in an increase in strength at a later age. In general, raising the content of calcium sulfate results in a lower strength of the concrete [15], while its presence speeds up the hydration process of CSA cement [16]. Hence, it is expected to attain higher strength at early ages by the addition of calcium sulfate to the CSA cement. However, it should be noted that the addition of more than 25% calcium sulfate can cause reduced strength due to cracking of the cement matrix. Dimensional stability features of CSA cement are significantly influenced by the variation of the gypsum and anhydrite content that were added to the raw mix [17]. The presence of gypsum (CaSO_4_) content between 18–20%, 22–24%, and higher than 25% resulted in slight shrinkage during setting, minimal dimensional change, and notable expansion, respectively [13].

Cement-based materials are weak materials in term of tensile strength, and thus concrete has a brittle behavior and cracks are practically unavoidable [18,19]. Cracks are created in different scales once the applied load exceeds the concrete’s tensile strength [20,21]. Additionally, the shrinkage of concrete can cause cracking in early stages and subsequently shortening of the serviceability of the concrete structure as a result of exposure to harmful substances [22]. The introduction of discrete fibers is accepted as a favorable solution in the manufacturing of composites with increased mechanical characteristics, because it controls the propagation of cracks [23,24,25,26,27]. Moreover, fibers can decrease drying shrinkage deformation either by improving cohesion between the concrete matrix and fibers, which contributes to physically restraining shrinkage [28], or controlling cracks (the most important effect of fibers in the shrinkage of concrete [29]). Nonetheless, the efficiency of fibers in concrete vary, depending on important factors such as fiber type, elastic modulus of the fiber, and fiber aspect ratio [30].

Despite the advantages of concrete compared to other construction materials, its brittleness can shorten the service life of structures. Moreover, drying shrinkage is critical for large surface area concrete structures and can decrease the overall strength and durability of concrete due to the formation of cracks. The objective of this study is to explore the impact of different fibers on the engineering properties of concretes manufactured with CSA cement. The properties of CSA cement-based concretes and particularly their hydration products have been assessed by other researchers [31,32,33]. Additionally, fiber-reinforced concrete (FRC) was developed as a material with enhanced ductility and durability features [34,35,36] and its mechanical and durability properties have been intensively investigated by other researchers. To the best of the authors’ knowledge, there is no previously published work investigating the effect of different fibers on the features of CSA cement-based concrete. Double hooked-end steel fibers, hooked-end steel fibers, and polyvinyl alcohol fibers were utilized with fiber content of 1%. Eight concrete mixes were manufactured at two water-cement ratios of 0.35 and 0.28. The mechanical properties and dimensional stability of the concrete mixes were assessed at various curing ages. Moreover, scanning electron microscopy (SEM) was used to explore the microstructural properties of concrete. The findings of this research are highly promising and show that introducing DHE steel fibers can substantially increase the engineering properties of CSA cement-based composite.

## 2. Materials and Methods

### 2.1. Characteristics of Materials

Calcium sulfoaluminate cement (CSA) produced by Italcementi Group (Bergamo, Italy),) was utilized in this study. The chemical composition and physical properties of CSA cement are listed in Table 1.

Silica fine aggregate and coarse aggregate were employed at 50.3% and 49.7%, respectively, to assure a uniform concrete. The features of the aggregates are presented in Table 2.

**Figure 1:**
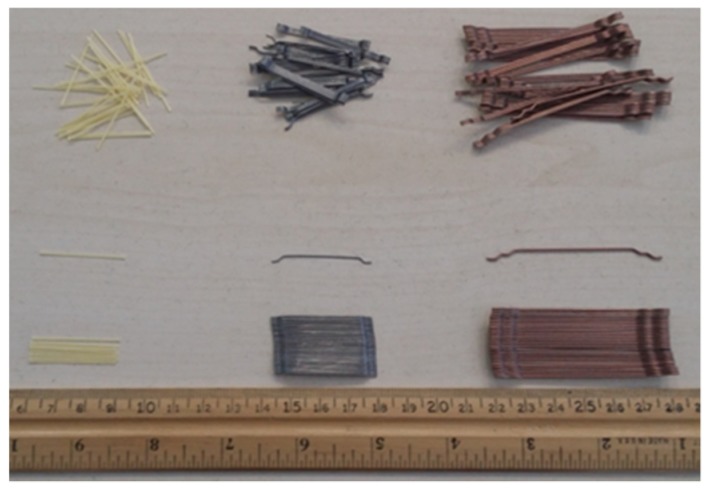
Shape and dimension of steel and polyvinyl alcohol fibers.

A Driver Care 10-Sika as a superplasticizer agent and tartaric acid as a retarder were utilized to adjust the workability and setting time of the concrete mixes. Double hooked-end (DHE) steel fibers with aspect ratio of 65, hooked-end (HE) steel fiber with aspect ratio of 65, and polyvinyl alcohol fiber (PVA) with aspect ratio of 75 were employed in this study. The appearance of different fibers is shown in Figure 1, and the features of fibers are given in Table 3.

### 2.2. Concrete Mix Proportions and Mixing Procedure

The absolute volume method [37] was used to design the concrete mixes, which were prepared at two water-cement ratios of 0.35, and 0.28. All the mixes were developed using a pan mixer. To avoid the absorption of aggregate moisture by the concrete mixer, the surface of the pan mixer was cleaned with a wet towel prior to adding the raw materials. The following mixing procedure was employed to produce a homogenous concrete. Fine materials, including sand, cement, and tartaric acid, were added to the mixer and mixed for one min. The water was split into two portions and one of them was mixed with SP and introduced to the mixer. The mixing procedure was carried on for an additional two min. The coarse aggregates in Saturated Surface Dry (SSD) condition and the remaining mixing water was added, and the materials mixed for another 5 min. Fibers were added slowly to the mixer and mixing was carried on for another 5 min to create a homogenous fiber-reinforced concrete (FRC). The mix proportions of different mixes along with their slump values are presented in Table 4. In Table 4, the dosage of superplasticizer and tartaric acid is demonstrated as a percentage of the cement weight. The slump tests were conducted in accordance with ASTM C143 [38] to determine the workability of fresh concrete. The target for slump test was to attain a slump of 18 ± 2 cm. The dosage of SP for FRC in different water-cement ratios was kept constant to observe the effect of fibers on the rheology of concrete. The addition of fibers in concrete adversely affects the workability of the concrete. The steel molds with different sizes as specified with standard codes were used to mold the fresh concrete. A lime-saturated water container with temperature of 23 °C was used to cure the concrete samples. For each reported test data, three specimens were prepared and the average values were presented.

### 2.3. Testing Methods

**Figure 2:**
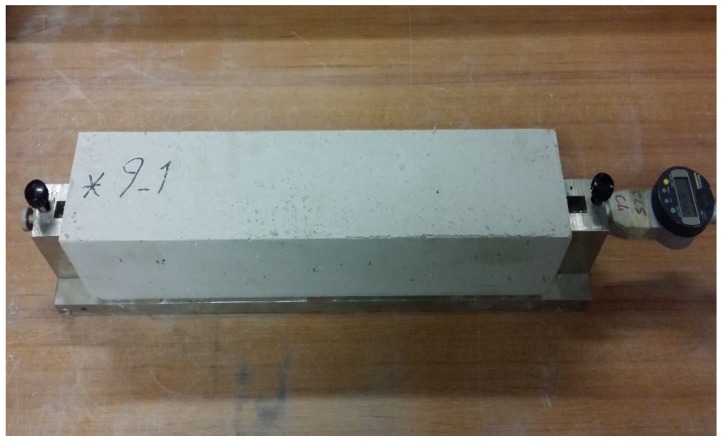
Test set up to measure the drying shrinkage of prismatic specimens.

Compressive strength tests were conducted on the cubic specimens with 100 mm dimension as per ASTM C39 [39]. The splitting tensile strength tests were carried out in accordance with ASTM C496 [40] by using cylindrical specimens with dimension of 100 mm × 200 mm. The elastic modulus of concrete specimens was assessed by using cylindrical specimens with diameter of 100 mm and height of 200 mm as per ASTM C469 [41]. The flexural performance of concrete was evaluated by using 150 mm × 150 mm × 600 mm beams in accordance with BS EN 14651 [42]. Dimensional stability under a drying condition was measured on 100 mm × 100 mm × 500 mm prismatic beams. The length change measurements were started after demolding of the specimens and continued at the ages of 1, 2, 3, 7, 14, 28, and 56 days. Dimensional stability was conducted on the specimens stored in a room with 50% RH level and temperature of 23 °C. The test set up for measuring the shrinkage of specimens is shown in Figure 2. The microstructure of concrete was studied in the secondary electron (SE) mode by means of the SEM method. A VEGA-II TESCAN microscope (TESCAN, Brno, Czech Republic) was used to explore the morphology of the concrete specimens. Carbon coating was used to avoid the charging problem.

## 3. Results and Discussions

### 3.1. Compressive Strength

**Figure 3:**
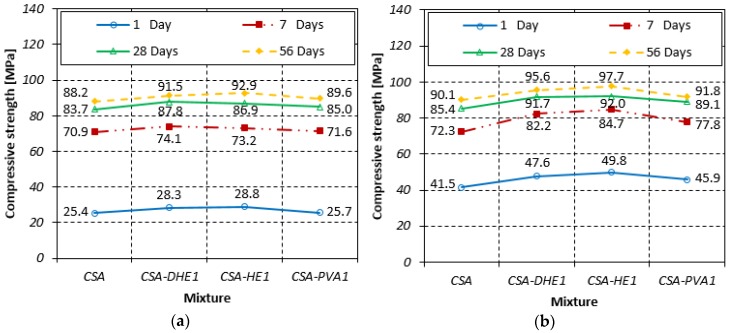
Compressive strengths of different fiber-reinforced concretes at water-cement ratios of: (**a**) 0.35, (**b**) 0.28.

The results of compressive strength tests for concrete developed at water-cement ratios of 0.35 and 0.28 are shown in Figure 3. The results indicate that the compressive strength of concrete was increased by introducing any type of fiber. The improved compressive strength of FRC can be attributed to the fibers’ capacity to control crack propagation, and reduced rate of crack development [43]. It was observed that the compressive strength of the specimens was developed at a water-cement ratio of 0.35, an increase from 1% to 13% as a result of the inclusion of fibers to the CSA mix. It can also be observed that steel fibers were more effective as compared to PVA fibers in increasing the strength of concrete. This can be attributed to the fact that steel fibers have a higher modulus of elasticity and tensile strength with respect to the PVA fibers that importantly control the extension of macro-cracks and improve compressive strength. The highest compressive strength at 56 days (i.e., 92.9 MPa) was achieved by the inclusion of 1.0% HE steel fiber in concrete. The positive influence of steel fibers on the compressive strength of FRC is reported by Song and Hwang [44]. It was observed that compressive strength was improved by 15.3% by adding 1.5% steel fiber content. Figure 3 shows that a similar trend occurred in the compressive strength of concrete specimens fabricated at water-cement ratio of 0.28. The results indicate that the introduction of fibers cause an increment in the compressive strength of FRC, irrespective of fiber type. This improvement ranged from 2% to 20%, depending on fiber type and testing age. The compressive strength of 97.7 MPa was achieved at 56 days by the CSA-HE1 mix, which showed the best performance among all the mixes. The scattering of data in terms of coefficient of variation ranged from 2.6% to 5.2%, depending on the water-cement ratio, fiber content, and testing age.

The results further demonstrate that strength development in concretes produced at a higher water-cement ratio (i.e., 0.35) was higher compared to that of concretes manufactured at a water-cement ratio of 0.28. For instance, the average 28 and 56-day compressive strengths of FRC with water-cement ratio of 0.35 were 19% and 25% higher than their 7-day strength, while the increase was 12% and 16% in FRC produced at a water-cement ratio of 0.28. This can be explained by the fact that the free water that was available in concretes with a water-cement ratio of 0.28 was consumed very fast by ye’elimite to generate ettringite, and a lesser amount of water was available for its further hydration and strength gain. On the other hand, in concretes that were manufactured at a water-cement ratio of 0.35, further reactions occurred at later ages, which consequently developed higher strength growth.

**Figure 4:**
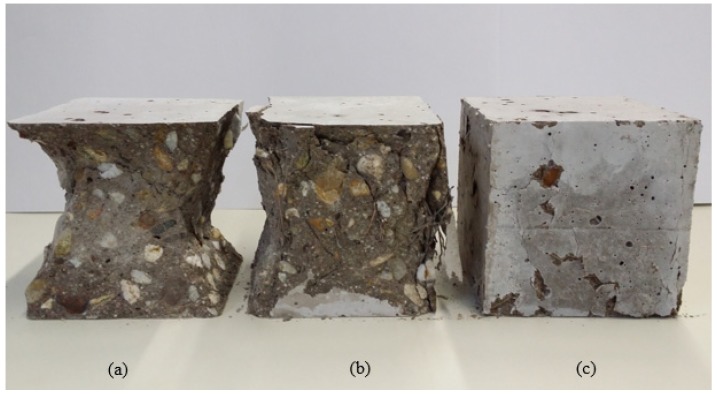
Failure mode of high performance concrete under compression load: (**a**) 0% fiber, (**b**) 1% DHE steel fiber, (**c**) 1% PVA fiber.

The failure mode of high performance concrete under compression loads is shown in Figure 4. As can be seen, the plain CSA specimen was significantly damaged and its behavior was almost explosive. However, the inclusion of fibers importantly changed the failure pattern of concrete. It was noticed that the PVA fibers act better than DHE steel fibers to restrain the propagation of micro-cracks in the concrete due to their higher number of fibers per volume of concrete at a similar fiber content.

### 3.2. Splitting Tensile Strength

**Figure 5:**
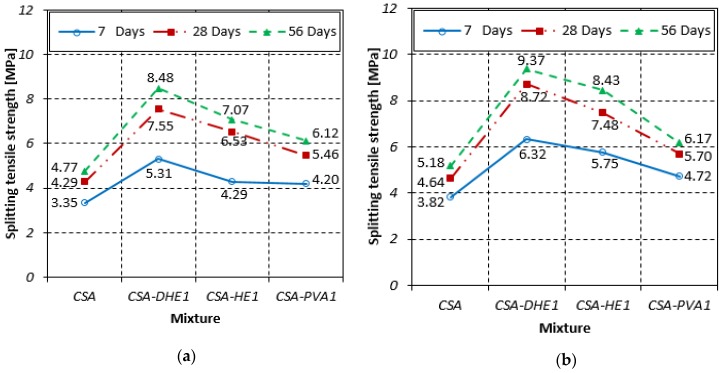
Splitting tensile strengths of different fiber-reinforced concretes at water-cement ratios of: (**a**) 0.35, (**b**) 0.28.

The splitting tensile strength results of different FRC mixes at water-cement ratios of 0.35 and 0.28 are shown in Figure 5. The results of FRC reveal that introducing fibers, particularly DHE steel fibers, can significantly improve the splitting tensile strength of the concrete. For example, the splitting tensile strength of the concrete mixes developed at a water-cement ratio of 0.35 and reinforced with 1% DHE steel fibers increased by 59%, 76%, and 78% at 7, 28, and 56 days, respectively, when compared with those of the reference CSA concrete. The improvement in the splitting tensile strength of CSA-HE1 mix ranged from 28% to 52%, while this increase for CSA-PVA1 mix varied from 25% to 28% as compared to those of the CSA mix, depending on the testing age. The tensile strength and modulus of elasticity of PVA fibers are lower compared to those of the steel fibers and PVA fibers mostly contribute to the controlling of micro-cracks. Therefore, the influence of PVA fibers on the splitting tensile strength is not comparable to that of the steel fibers. Furthermore, DHE steel fibers utilized in the current study created remarkably increased pullout forces with respect to other fibers due to the anchoring mechanism, which led to an improved tensile strength [26]. Noushini et al. [45] also investigated the influence of PVA fibers with different lengths and at various fiber contents. It was reported that the improvement in splitting tensile strength varied between 11% and 32.5%, depending on fiber length and content.

For FRC developed at a water-cement ratio of 0.28, a similar trend to concretes with a water-cement ratio of 0.35 was observed. The addition of fibers in concrete caused an improvement in splitting tensile strength, irrespective of the type of fibers. As can be seen in Figure 5, the highest splitting tensile strength was attained once 1% DHE steel fibers was employed. For instance, the splitting tensile strength of this mix increased by 65%, 88%, and 81% at 7, 28, and 56 days, respectively, over that of the plain CSA concrete. It was observed that the increase in the splitting tensile strength of CSA-HE1 mix ranged from 51% to 63%, while this increase for the CSA-PVA1 mix varied from 19% to 24%, as compared to those of the CSA mix, depending on the testing age. The coefficient of variation of splitting tensile strength test results varied between 3.3% and 7.4%, depending on the water-cement ratio, fiber content, and testing age.

The results further demonstrate that the curing of specimens had a higher impact on the improvement of FRC strength when compared to that of the plain CSA concrete. For example, the average 28- and 56-day splitting tensile strengths of FRC with water-cement ratio of 0.35 were 42% and 57% higher than their 7-day strength. This suggests that the chemical cohesion between fibers and cement matrix was increased over time because of cement expansion, and consequently caused an increment in the splitting tensile strength of the concrete. The impact of curing age on the enhancement of splitting tensile strength of concrete was reduced by decreasing the water-cement ratio. For example, the average 28- and 56-day splitting tensile strengths of FRC with a water-cement ratio of 0.28 were 30% and 42% higher than their splitting tensile strength at seven days. The reason is that in concretes with low water-cement ratio (i.e., 0.28), free water was consumed significantly in the early periods and developed higher strength at seven days. Therefore, there is a lack of free water inside the concrete for further cement hydration at later periods (i.e., 28 and 56 days).

### 3.3. Modulus of Elasticity

**Figure 6:**
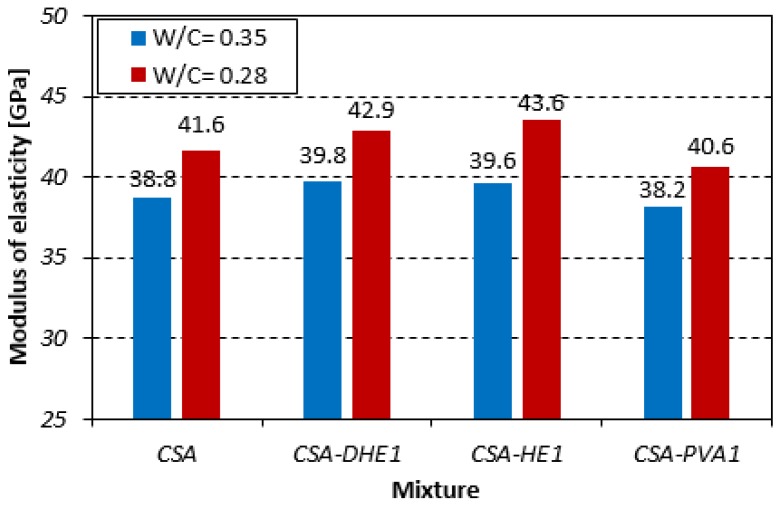
Modulus of elasticity of different fiber-reinforced concretes.

The 28-day modulus of elasticity of different FRC mixes with water-cement ratios of 0.35 and 0.28 is shown in Figure 6. The results reveal that the inclusion of metallic fibers resulted in a negligible increase in the elastic modulus of concrete, while introducing PVA fibers in concrete slightly reduced its modulus of elasticity. The improved modulus of elasticity of concretes with steel fibers can be attributed to the influence of steel fibers on the stiffness of the composite. The coefficient of variation of modulus of elasticity test results varied between 1.9% and 4.8%, depending on the water-cement ratio, fiber content, and testing age. The influence of fiber types on the modulus of elasticity of self-compacting concrete was investigated by Beigi et al. [46]. It was revealed that the introduction of fibers had an insignificant impact on the elastic modulus of concrete. Their results showed that the modulus of elasticity of FRC was reduced slightly in some cases, when compared to that of the reference concrete. This is in good agreement with the results obtained in the current study.

### 3.4. Flexural Behavior

#### 3.4.1. Flexural Load-CMOD Curves

**Figure 7:**
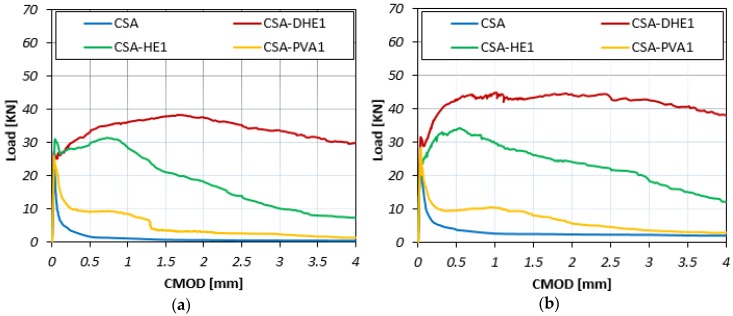
Flexural load-CMOD curves of different fiber-reinforced concretes cured for seven days at water-cement ratios of: (**a**) 0.35, (**b**) 0.28.

**Figure 8:**
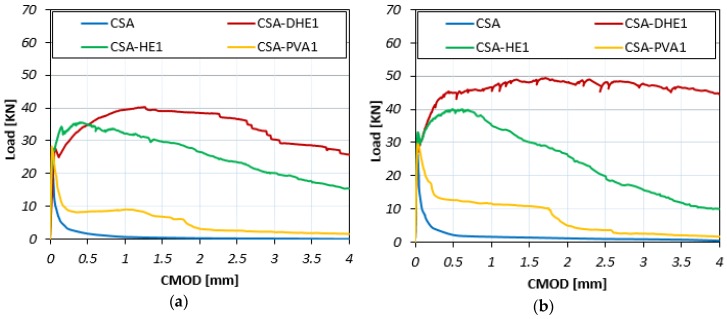
Flexural load-CMOD curves of different fiber-reinforced concretes cured for 28 days at water-cement ratios of: (**a**) 0.35, (**b**) 0.28.

**Figure 9:**
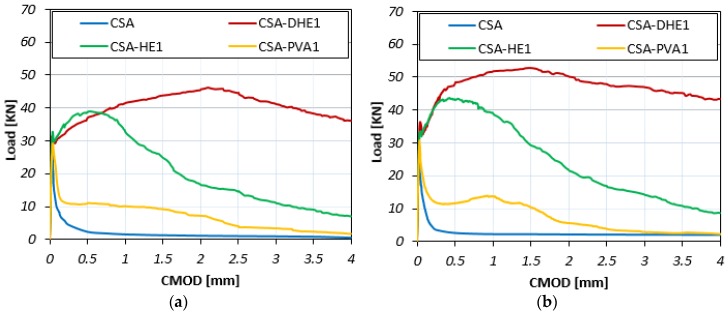
Flexural load-CMOD curves of different fiber-reinforced concretes cured for 56 days at water-cement ratios of: (**a**) 0.35, (**b**) 0.28.

The Load-CMOD curves for different fiber-reinforced concretes at curing ages of 7, 28, and 56 days are illustrated in Figure 7, Figure 8 and Figure 9. The results reveal that the inclusion of fibers led to an increment in the maximum flexural load of concrete at all curing ages irrespective to the fiber type. For example, the flexural strength improvement of CSA-DHE1, CSA-HE1, and CSA-PVA1 mixes ranged from 55% to 73%, 37% to 42%, and 7% to 15%, respectively, depending on the age of testing. As can be noticed, the concretes containing 1% DHE steel fibers exhibited the highest flexural strength compared to other FRC considered in this study. The anchoring mechanism, high tensile strength, and high modulus of elasticity of DHE steel fibers are the main factors that importantly contribute to the improved flexural strength of CSA-DHE1 mix. Simões et al. [47] demonstrated that concrete samples were reinforced with DHE steel fibers achieved significantly higher peak load in fiber pull-out test as compared to the conventional steel FRC, and because of that the flexural strength of concrete can be substantially improved. The results further indicate that the Load-CMOD behavior of concretes is significantly different, depending on the fiber type used. As can be observed in Figure 7, Figure 8 and Figure 9, introducing 1% DHE steel fibers in CSA concrete resulted in a deflection-hardening behavior in concrete, while concretes reinforced with 1% HE steel fibers exhibited a deflection-softening performance. The results also demonstrate that the CMOD matching the maximum flexural load varied from 1.26 mm to 2.11 mm for the CSA-DHE1 mix, while this varied from 0.39 mm to 0.72 mm for the CSA-HE1 mix, depending on the testing age. This can be attributed to the capacity of DHE steel fibers to restrain the wide spreading of macro-cracks in concrete as a result of its higher length, tensile strength, and anchoring mechanism. On the contrary, HE steel fibers due to their lower tensile strength and length improved the flexural load carrying capacity of concrete beyond the appearance of the first crack, up to CMOD equal to 0.72, and caused a deflection-softening behavior due to extensive cracks creation. Moreover, the results show that the inclusion of PVA fibers in concrete slightly increased the flexural load and after the appearance of the first crack, PVA fibers were not able to connect the macro-cracks and prevent further crack propagation. As a consequence, the flexural load significantly reduced and an almost flat behavior in load carrying capacity of the CSA-PVA1 mix was seen.

In general, a trend similar to the flexural behavior of concretes was developed at a water-cement ratio of 0.35 for concrete mixes with a water-cement ratio of 0.28. The addition of fibers increased the flexural strength of concrete, irrespective of fiber type. For example, the improvement in flexural strength of CSA-DHE1, CSA-HE1, and CSA-PVA1 mixes ranged from 86% to 92%, 47% to 56%, and 10% to 20%, respectively, depending on the testing age. Similarly, a deflection-hardening behavior was achieved in the CSA-DHE1 mix, while the addition of other kinds of fibers led to a deflection-softening behavior. The results indicate that the highest flexural strength was achieved by the CSA-DHE1 mix—its flexural strength at 56 days was 16.9 MPa.

#### 3.4.2. Residual Flexural Tensile Strength

**Figure 10:**
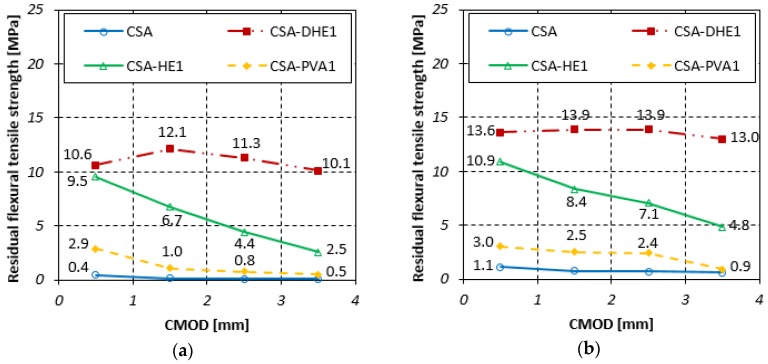
Residual flexural tensile strength of different fiber-reinforced concretes cured for 7 days at water-cement ratios of: (**a**) 0.35, (**b**) 0.28.

**Figure 11:**
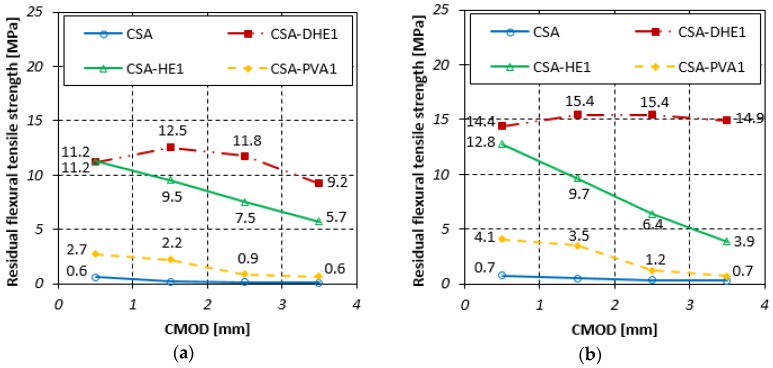
Residual flexural tensile strength of different fiber-reinforced concretes cured for 28 days at water-cement ratios of: (**a**) 0.35, (**b**) 0.28.

**Figure 12:**
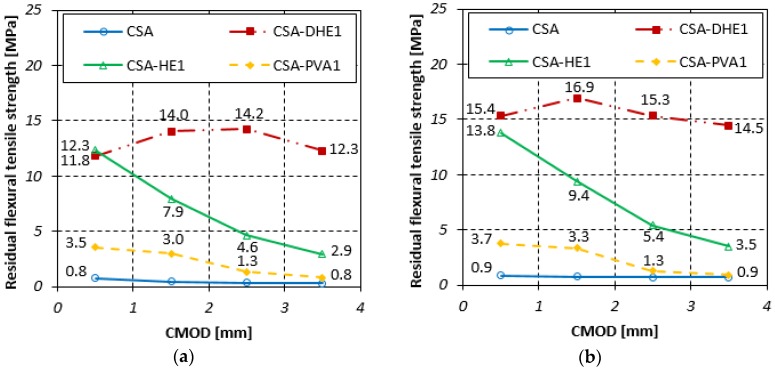
Residual flexural tensile strength of different fiber-reinforced concretes cured for 56 days at water-cement ratios of: (**a**) 0.35, (**b**) 0.28.

The residual flexural tensile strengths for different FRC at curing ages of 7, 28, and 56 days are illustrated in Figure 10, Figure 11 and Figure 12. Fr_1_, Fr_2_, Fr_3_, and Fr_4_ show residual flexural strengths matching CMOD at 0.5 mm, 1.5 mm, 2.5 mm, and 3.5 mm, respectively. Generally, it can be seen that an increment in CMOD results in a decrease in residual flexural tensile strengths. However, the flexural performance of CSA-DHE1 is different from other mixes and a deflection-hardening behavior was observed. The results indicate that Fr_2_ is the highest residual flexural tensile strength in the mix containing 1% DHE steel fibers, and a reduction took place in Fr_3_ and Fr_4_. For instance, the Fr_2_ and Fr_3_ of the CSA-DHE1 mix cured for 28 days increased by 12% and 5% as compared to its Fr_1_, while the Fr_4_ reduced by 18% as compared to its Fr_1_. The results of the CSA-HE1 mix at 28 days indicate that the Fr_2_, Fr_3_, and Fr_4_ reduced by 15%, 33%, and 49%, respectively as compared to its Fr_1_. These results approve the important effect of DHE steel fibers in enhancing the flexural behavior of concrete and can undoubtedly have a key helpful contribution in designing concrete structure subjected to bending load.

Similarly, the results of concrete prepared at a water-cement ratio of 0.28 indicate that the residual flexural tensile strengths of concrete containing 1% DHE steel fibers were significantly higher with respect to those of the concretes reinforced with 1% HE steel or PVA fibers, particularly at greater CMOD. For instance, the Fr_3_ of CSA-DHE1, CSA-HE1, and CSA-PVA1 mixes at 28 days were 15.4, 6.4, and 1.2 MPa, respectively. As can be noticed in Figure 10, Figure 11 and Figure 12, CSA-DHE1 were the only specimens at both water-cement ratios that show a deflection-hardening response and Fr_3_ and Fr_4_ were slightly lower than Fr_2_. This can be explained by the fact that the DHE steel fibers bridge the macro-cracks and absorb more energy as the flexural load exceeding the strength of concrete and its anchoring mechanism have an important influence on the improvement of flexural strength [48].

**Figure 13:**
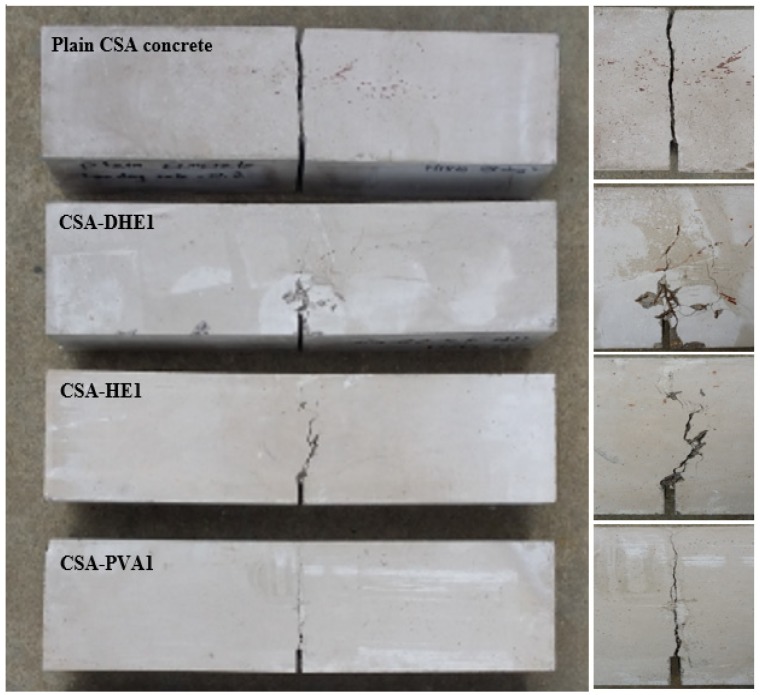
Crack propagation on the surface of concrete beams reinforced with different types of fibers (close-up of crack propagation is shown on the right side of each beam).

Crack propagation on the surface of concrete beams containing different types of fibers is illustrated in Figure 13. In addition, close-up of crack propagation is shown on the right side of each beam in the same figure. As can be observed, plain concrete without any kind of fiber was split into two parts as a result of exceeding the flexural load over the flexural strength of concrete. The results of FRC reveal that the inclusion of metallic fibers and particularly DHE steel fibers led to the appearance of multiple micro-cracks around a single big crack along the notch. Figure 13 shows that introducing 1% PVA fibers, although improved the flexural strength of concrete by restraining micro-cracks, was not able to further prevent the extension of macro-cracks and a single big crack occurred in this mix.

**Figure 14:**
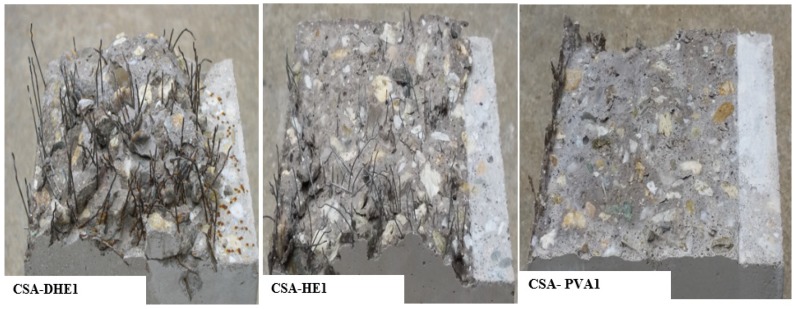
Cross section of fractured concrete beams reinforced with different types of fibers and distribution of fibers on their surfaces.

It should be noted that the orientation of fibers and their distribution in the concrete beams have an important effect on the flexural performance of concrete. The cross sections of fracture concrete beams containing different types of fibers and the distribution of fibers on their surface are shown in Figure 14. As can be seen, DHE steel fibers were dispersed uniformly in the cross section of concrete and majority of the fibers were aligned perpendicular to the fracture surface. This can result in a good flexural performance, as already shown in the Load-CMOD curves. Figure 14 further shows that the HE steel fibers were not dispersed uniformly at the cross section of the concrete beams, and some area remained uncovered with the fibers. This may result in the variation of flexural behavior and adversely affect the post-cracking behavior of the specimens. The fractured surface of CSA-PVA1 mix shows that the PVA fibers were ruptured or debonded from the cement matrix. As PVA fibers are straight and have lower length and tensile strength with respect to those of the metallic fibers, they are not able to significantly increase the flexural strength of concrete and cause an improvement in the flexural toughness of concrete.

### 3.5. Dimensional Stability

**Figure 15:**
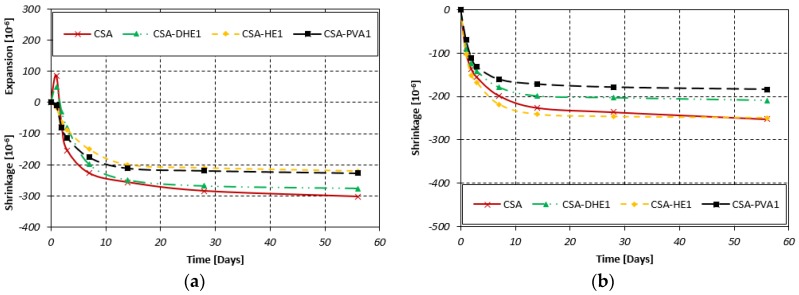
Dimensional stability of different fiber-reinforced concretes at water-cement ratios of: (**a**) 0.35, (**b**) 0.28.

The results of the dimensional stability test under drying conditions for different fiber-reinforced concretes at water-cement ratios of 0.35 and 0.28 are shown in Figure 15. The results of concretes with a water-cement ratio of 0.35 indicate that the addition of fibers in CSA concrete caused a reduction in the shrinkage stain, irrespective of fiber type. It was observed that the HE steel and PVA fibers fully canceled the expansion of concrete at day 1, while an expansion equal to 50 µm/m occurred in the mix containing 1% DHE steel fibers. The shrinkage strains of CSA-DHE1, CSA-HE1, and CSA-PVA1 mixes at 56 days were 276, 220, and 227 µm/m, respectively, reduced by 9%, 27%, and 25%, compared to that of the CSA mix. The higher efficiency of HE steel and PVA fibers in restraining the shrinkage of concrete can be explained by the fact that a higher number of fibers were available in cement composites, which resulted in volume stability of those concretes. Additionally, Passuelo et al. [49] reported that PVA fibers may reduce the free shrinkage of concrete by modifying the internal water movement within the concrete.

The results further indicate that contrary to concretes manufactured at a higher water-cement ratio (i.e., 0.35), no evidence of expansion occurred in CSA-based mixes at a water-cement ratio of 0.28. In other words, the expansion of concrete at day 1 has been fully canceled and a greater amount of shrinkage took place in the early periods as compared to concretes with a water-cement ratio of 0.35. This can be explained by the appearance of autogenous shrinkage that occurred in these concretes as a result of higher cement content and lower water-cement ratio. However, the shrinkage strain of concretes at later periods (i.e., 56 days) was lower as compared to concrete with a higher water-cement ratio. Cheung and Leung [50] studied the autogenous and drying shrinkage of concrete produced at different water-binder ratios of 0.19, 0.3, and 0.4. They reported that the autogenous shrinkage represented a significant proportion of the total shrinkage in the concrete with a water-binder ratio of 0.19, whereas this was highly reduced in concretes with higher water-binder ratios of 0.3 and 0.4. The results of fiber-reinforced concretes show that the addition of discrete fibers caused a reduction in shrinkage strain. As can be seen in Figure 15, the lowest shrinkage deformation was developed by the mix containing 1% PVA fibers—it was reduced by 27% as compared to that of the plain CSA mix. This was followed by the CSA-DHE1 and CSA-HE1 mixes that attained a shrinkage strain of 209 µm/m and 249 µm/m, respectively, while the shrinkage of reference CSA mix was 252 µm/m. The findings of this study are in close agreement with previous studies that reported that fibers can control shrinkage cracking [51,52].

### 3.6. SEM Observations

**Figure 16:**
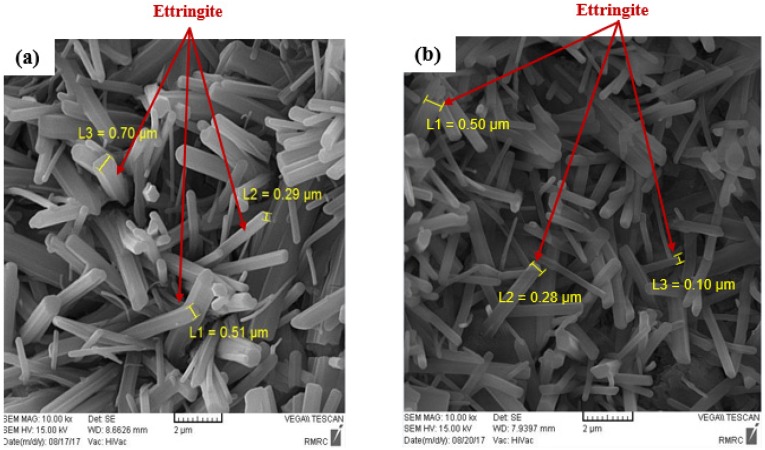
SEM images of CSA cement-based concretes at water-cement ratios of: (**a**) 0.35, (**b**) 0.28.

SEM observation was performed in the secondary electron (SE) mode to study the microstructure of concrete; the images are shown in Figure 16, which shows the formation a rich amount of ettringite in CSA cement-based concretes at both water-cement ratios as a result of ye’elimite hydration. The results reveal that the size of prismatic ettringite crystals in the CSA mix with a water-cement ratio of 0.35 ranged from 0.29–0.7 µm wide with 2–6 µm length. The presence of a high amount of prismatic ettringite crystals in CSA concrete can be responsible for its higher mechanical properties strength and also lead to dimension stability of concrete [53,54]. The results further indicate that the size of ettringite crystals was reduced by decreasing the water-cement ratio. In concrete with a lower water-cement ratio, the size of prismatic ettringite crystals varied between 0.1–0.5 µm wide, and 2–6 µm length.

**Figure 17:**
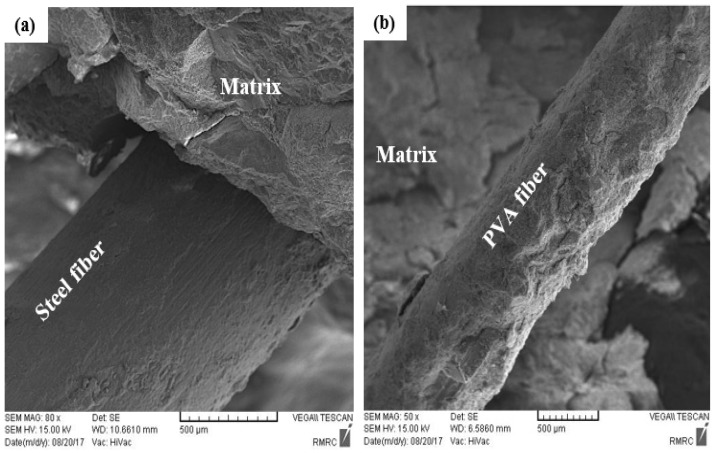
SEM images of interface between fibers and cement paste: (**a**) steel fibers, (**b**) PVA fibers.

Figure 17 shows the SEM observation performed on the FRC to explore the bond between the fibers and cement matrix. As can be seen in Figure 17a, there is a good bond between the metallic fiber and cement matrix, and a significant amount of hydrated cement adhered to the surface of the steel fiber. Similarly, Figure 17b shows that a great amount of cement covered the surface of the PVA fibers. This result confirms that the inclusion of CSA cement in concrete improves the chemical cohesion between fibers and cement matrix as a result of cement expansion.

## 4. Conclusions

This paper evaluated the effect of different types of fibers on the engineering properties of CSA cement-based concretes. The following conclusions are presented from the experimental results:

1. The results indicate that the addition of fibers causes an increase in the compressive strength of concrete, irrespective of fiber type. The compressive strength improvement of FRC at a water-cement ratio of 0.35 varied from 1% to 13%, while this improvement at a water-cement ratio of 0.28 ranged from 2% to 20%, depending on fiber type and testing age.
2. The inclusion of 1% fibers and particularly DHE steel fibers in CSA cement-based mixes results in an increase in the splitting tensile and flexural strength of FRC. Introducing DHE steel fibers in CSA concrete results in a deflection-hardening behavior in concrete, while concretes reinforced either with HE steel fibers or PVA fibers exhibit a deflection-softening response. The improved post-cracking behavior of the FRC can be explained by the fact that the DHE steel fibers inhibit the development of macro-cracks through the anchoring mechanism.
3. The addition of fibers in concrete has no important influence on the elastic modulus of FRC.
4. Introducing discrete fibers in CSA cement-based concretes reduces the shrinkage deformation of FRC, irrespective of the water-cement ratio and fiber type. CSA-PVA1 shows the lowest shrinkage among different mixes and its final shrinkage was decreased by 25% and 27% at water-cement ratios of 0.35 and 0.28, respectively.
5. The prismatic ettringite crystals with 0.29–0.7 µm wide, and 2–6 µm length are the primary hydration products of CSA mix with a water-cement ratio of 0.35. The results further indicate that the size of ettringite crystals is reduced by decreasing the water-cement ratio, and crystals with 0.1–0.5 µm wide, and 2–6 µm length were developed. The SEM results also confirms that the chemical cohesion between the cement matrix and fibers has been improved.

## Figures and Tables

**Table 1:** Chemical composition and physical properties of CSA cement.

**Oxide Compositions**	**SiO_2_**	**Al_2_O_3_**	**Fe_2_O_3_**	**MgO**	**SO_3_**	**Na_2_O**	**K_2_O**	**CaO**	**Specific Gravity (g/cm^3^)**	3.1
Weight %	7.5	35.0	1.5	--	15.5	--	--	41.0		
**Mineralogical Phase Compositions**	**C_2_S**	**C_4_A_3_$**		**C_11_A_7_F**	**CaSO_4_**	**Fluorellestad**		**Others**	**Specific Surface (m^2^/kg)**	450
Weight %	18.0	60.0		4.4	9.9	4.5		3.2		

**Table 2:** Physical properties of the aggregates.

Aggregate Type	Maximum Size Aggregate (mm)	Water Absorption (%)	Specific Gravity	Fineness Modulus
Fine aggregate	4.75	1.1	2.65	2.9
Coarse aggregate	19.0	0.96	2.74	-

**Table 3:** Properties of hooked-end steel and PVA fibers.

Type and Shape of Fiber	Length *l* (mm)	Diameter *d* (mm)	Aspect Ratio *l/d*	Density (g/cm^3^)	Tensile Strength (N/mm^2^)
Double hooked-end steel (DHE)	60	0.9	65	7.8	2300
Hooked-end steel (HE)	35	0.55	65	7.8	1050
Polyvinyl alcohol (PVA)	30	0.4	75	1.3	900

**Table 4:** Mix proportions of fiber-reinforced concrete mixes.

Mixture ID	W/C	Water	CSA	FA	CA	Fiber Volume Fraction (%)			SP (%)		Slump (cm)
		(kg/m^3^)				DHE	HE	PVA	DC10	TA	
CSA	0.35	157.5	450	901	891	-	-	-	1.2	0.2	20
CSA-DHE1	0.35	157.5	450	888	878	1	-	-	1.4	0.2	19
CSA-HE1	0.35	157.5	450	888	878	-	1	-	1.4	0.2	18
CSA-PVA1	0.35	157.5	450	888	878	-	-	1	1.4	0.2	15
											
CSA	0.28	154.0	550	863	853	-	-	-	1.5	0.2	18
CSA-DHE1	0.28	154.0	550	849	840	1	-	-	1.7	0.2	20
CSA-HE1	0.28	154.0	550	849	840	-	1	-	1.7	0.2	18
CSA-PVA1	0.28	154.0	550	849	840	-	-	1	1.7	0.2	16

CSA: calcium sulfoaluminate cement; FA: fine aggregate; CA: coarse aggregate; DHE: double hooked-end steel fiber; HE: hooked-end steel fiber; PVA: polyvinyl alcohol fiber; DC10: Driver Care 10-Sika; TA: tartaric acid.
